# Environmental Toxin Screening Using Human-Derived 3D Bioengineered Liver and Cardiac Organoids

**DOI:** 10.3389/fpubh.2018.00103

**Published:** 2018-04-16

**Authors:** Steven D. Forsythe, Mahesh Devarasetty, Thomas Shupe, Colin Bishop, Anthony Atala, Shay Soker, Aleksander Skardal

**Affiliations:** ^1^Wake Forest School of Medicine, Wake Forest Institute for Regenerative Medicine, Medical Center, Winston-Salem, NC, United States; ^2^Virginia Tech-Wake Forest School of Biomedical Engineering and Sciences, Wake Forest School of Medicine, Medical Center Boulevard, Winston-Salem, NC, United States; ^3^Comprehensive Cancer Center at Wake Forest Baptist Medical, Medical Center Boulevard, Winston-Salem, NC, United States; ^4^Department of Cancer Biology, Wake Forest School of Medicine, Medical Center Boulevard, Winston-Salem, NC, United States

**Keywords:** tissue engineering and regenerative medicine, organoids, environmental toxins, hepatocytes, cardiomyocytes, three dimensional

## Abstract

**Introduction:**

Environmental toxins, such as lead and other heavy metals, pesticides, and other compounds, represent a significant health concern within the USA and around the world. Even in the twenty-first century, a plethora of cities and towns in the U.S. have suffered from exposures to lead in drinking water or other heavy metals in food or the earth, while there is a high possibility of further places to suffer such exposures in the near future.

**Methods:**

We employed bioengineered 3D human liver and cardiac organoids to screen a panel of environmental toxins (lead, mercury, thallium, and glyphosate), and charted the response of the organoids to these compounds. Liver and cardiac organoids were exposed to lead (10 µM–10 mM), mercury (200 nM–200 µM), thallium (10 nM–10 µM), or glyphosate (25 µM–25 mM) for a duration of 48 h. The impacts of toxin exposure were then assessed by LIVE/DEAD viability and cytotoxicity staining, measuring ATP activity and determining IC50 values, and determining changes in cardiac organoid beating activity.

**Results:**

As expected, all of the toxins induced toxicity in the organoids. Both ATP and LIVE/DEAD assays showed toxicity in both liver and cardiac organoids. In particular, thallium was the most toxic, with IC50 values of 13.5 and 1.35 µM in liver and cardiac organoids, respectively. Conversely, glyphosate was the least toxic of the four compounds, with IC50 values of 10.53 and 10.85 mM in liver and cardiac organoids, respectively. Additionally, toxins had a negative influence on cardiac organoid beating activity as well. Thallium resulting in the most significant decreases in beating rate, followed by mercury, then glyphosate, and finally, lead. These results suggest that the 3D organoids have significant utility to be deployed in additional toxicity screening applications, and future development of treatments to mitigate exposures.

**Conclusion:**

3D organoids have significant utility to be deployed in additional toxicity screening applications, such as future development of treatments to mitigate exposures, drug screening, and environmental toxin detection.

## Introduction

Environmental toxins continue to represent a significant health concern within the United States and around the world. Even in the twenty-first century, we continue to hear of cities and towns in the U.S. have suffered from exposures to lead in drinking water and other heavy metals in food or the earth (1, 2). Moreover, there is equal potential for other locations to suffer such exposures in the future due to possible deregulation of those government agency policies that manage environmental toxins and other compounds.

Environmental heavy metals have long been known to cause cell and tissue damage, often leading to major health complications, especially in children (3). Exposure to heavy metals has been implicated in a host of gastrointestinal, hepatic, cardiac, and neurological disorders, including autism spectrum disorder (4). Increasing industrial and agricultural uses for heavy metals over the span of many years have led to an exponential increase in the concentration of these materials in soil and water, and subsequently in a variety of food sources, resulting in passage to humans through a number of methods, including consumption, inhalation, and direct skin contact (4, 5). Within the human body, environmental heavy metals have been demonstrated to affect the functionality of cell organelles, lipid membranes, and cytoplasmic enzymes. Additionally, heavy metals are able to interact directly with nucleic acids and nuclear proteins resulting in DNA damage leading to altered cell function, cancer, and cell death (6, 7). In addition to heavy metals, some compounds in pesticides have been determined to cause toxic effects in humans and wildlife, with many becoming banned due to these consequences, the most famous being DDT (8, 9). In addition to cases of acute toxicity, possible negative chronic afflictions on exposed populations include cancers, diabetes, endocrine disruption, neurodegenerative diseases, and potential epigenetic changes (10–15).

In the context of environmental toxin research, few if any significant studies have employed truly human-representative microphysiological systems (16, 17), such as 3D organoids or bioengineered tissue constructs, to assess the effects of environmental toxins on human physiology. Many studies do exist that employ traditional 2D cull cultures or animal studies, neither of which is necessarily completely representative of human physiology or human response to drugs and other compounds. Despite useful for many areas of research, animal models allow limited manipulation and deep study of biological mechanisms at play (18). On the other hand, traditional *in vitro* 2D cultures fail to recapitulate the 3D microenvironment of *in vivo* tissues (19). Drug diffusion kinetics vary dramatically, drug doses effective in 2D are often ineffective when scaled to animals or human patients, and cell–cell/cell–matrix interactions are inaccurate (20, 21). Moreover, the very nature of 2D versus 3D environments can significantly impact cellular behavior and phenotype. For example, in studies on cancer and mimicking the tumor microenvironment, our group recently demonstrated that on 2D tissue culture dishes, metastatic cancer cells appeared epithelial, not malignant, but when transitioned into a 3D tissue organoid environment they “switched” to a more accurate mesenchymal and metastatic phenotype (22, 23). Such 3D models have rapidly gained favor in areas, such as cancer and drug development, yet deployment for applications such as environmental toxin research has lagged as few examples exist in the literature. Yet 3D organoids could be valuable tools in environmental toxicity and safety research. These types of technologies could be used for a variety of applications, ranging from diagnostic testing of materials collected in field studies to development of treatment regimens—such as chelation therapies in the case of some heavy metal exposures—that can be used in human patients.

Our group has developed 3D organoids for multiple organs with human-like physiological function consisting of either primary cell or induced pluriopotent stem (iPS) cell origin for the purpose of testing compounds for efficacy and safety. Notably, these organoids are comprised of representative cell populations from their *in vivo* counterparts, can be maintained with high viability for long periods of time, are metabolically active, and respond to and metabolize drugs accurately (24, 25). Here, we describe the utilization of these organoids for the testing of environmental toxins on two organs often implicated in environmental toxicity—cardiac and liver. We performed a series of tests to assess the negative effects on both cardiac and liver organoids for each environmental toxin, including three heavy metals and one pesticide active component, with the rationale of creating a 3D *in vitro* system with a high similarity toward the parent tissue. Our results suggest the utility of our system allows for rapid testing of potential environmental hazards with the ability to induce large-scale reproducibility and find precise estimation of toxicity levels in multiple organ systems.

## Materials and Methods

No human subjects were employed in the described studies, as all cells were purchased commercially. Additionally, all experiments involving biohazards, select agents, and toxins were performed in line with institutional safety guidelines of Wake Forest Baptist Medical Center.

### Liver and Cardiac Cell Sources, Culture, and Organoid Formation

For liver organoids, all cells used were commercially sourced, human primary cells. Hepatic stellate cells (HSCs) (ScienCell, Carlsbad, CA, USA) were expanded in culture for two passages before cryopreservation for use in organoid formation. During expansion, HSCs were cultured in 90% high glucose DMEM (Thermo Fisher, Waltham, MA, USA) and 10% fetal bovine serum (Atlanta Biologicals, Flowery Branch, GA, USA) on a rat tail collagen I coating (10 g/cm^2^, Corning, Corning, NY, USA) at 37°C with 5% CO_2_ primary human hepatocytes (Triangle Research Labs, RTP, NC) were thawed according to manufacturer instructions using hepatocyte thawing medium (Triangle Research Labs). Kupffer cells were also thawed *via* manufacturer instructions (Gibco, Waltham, MA, USA). Primary human hepatocytes (Triangle Research Labs) were thawed as mentioned above, then plated on collagen coated (10 g/cm^2^, Corning) 6-well culture plates, using hepatocyte plating medium (Triangle Research Labs) at a density of ~150,000 cells/cm^2^. Cells were incubated at 37°C with 5% CO_2_ for 4 h before adding matrigel as an overlay (BD Biosciences, San Jose, CA, USA). Following further incubation for 24 h, fresh HCM medium (Lonza, Walkersville, MD, USA) was added.

For cardiac organoids, induced pluripotent stem cell-derived cardiomyocytes (iPSC CMs) were commercially sourced from Axiogenesis (cat. #COR.4U Cardiomyocytes). Human primary cardiac fibroblasts were commercially sourced from ScienCell (cat. #6330). Prior to organoid formation, iPSC CMs were cultured on tissue culture plastic for 48 h in COR.4U medium until cells began beating spontaneously. At this point, iPSC CMs were harvested using trypsin-EDTA (Hyclone, Logan, UT).

The liver cells were combined in a cell seeding mixture comprised of 90% HCM medium (Lonza), 10% heat-inactivated fetal bovine serum (Gibco), and rat tail collagen I (10 ng/µl, Corning). Liver organoids were produced with a mixture of 80% hepatocytes (Triangle Research Labs), 10% hepatic stellate cells (ScienCell), and 10% Kupffer cells (Gibco). Approximately 1,500 cells per 40 µL media were used to form aggregates in each well of a non-adherent, round-bottom, 96-well plates to produce spherical organoids (#7007, Corning). Cardiac organoids were produced similarly. IPSC CMs were suspended in cardiomyocyte maintenance medium (CMM, Stem Cell Theranostics, Redwood City, CA, USA). Fibroblasts were added as 10% of the total cell number, and the volume was adjusted to reach a cell density of 10,000 cells/mL. 100 µL of cell suspension was pipetted into each well of a non-adherent, round-bottom, 96-well plates to produce spherical organoids resulting in approximately 1,000 cells/organoid. Well plates were incubated and observed daily until organoid formation, and then immediately used in experiments.

### Preparation of Drug Stock Solutions

All four compounds were purchased from Sigma Aldrich (St. Louis, MO, USA). Drugs were dissolved in DI H_2_O for environmental toxins to reach 20 mM concentration for lead (II) chloride (203572), 10 mM for mercury (II) chloride (215465), and thallium nitrate (204609), and 50 mM concentration for glyphosate (45521), respectively. Doses were taken from previous research on toxicity in model systems and experiments were performed to determine an IC50 cellular activity for ATP assay. Serial dilutions were performed in media for each cell type until all concentrations were created at 2X final concentration. Specifically, lead was assessed at 10 μM–10 mM; mercury was assessed at 200 nM–200 µM; thallium was assessed at 10 nM–100 μM; and glyphosate was assessed at 25 μM–25 mM (Tables 1 and 2).

**Table 1:** Summary of the effects of the environmental toxins on liver spheroids.

Environmental toxins on liver organoids	Range of doses	Live/dead effective dose	ATP IC50
Glyphosate	25 µM–25 mM	250 µM–2.5 mM	10.53 mM
Lead	10 µM–10 mM	100 µM–1 mM	2.98 mM
Mercury	200 nM–200 µM	2–20 µM	30.8 µM
Thallium	10 nM–10 µM	1–10 µM	13.5 µM

*Effective dose for live/dead imaging is summarized as a qualitative measure of rapid cell death detected in images*.

**Table 2:** Summary of the effects of the environmental toxins on cardiac spheroids.

Environmental toxins on cardiac organoids	Range of Doses	Live/Dead Effective Dose	IC50	Heart beat effective dose at 30 min	Heart beat effective dose at 24 h	Heart beat effective dose at 48 h
Glyphosate	25 µM–25 mM	2.5–25 mM	10.85 mM	2.5 mM	2.5 mM	2.5 mM
Lead	10 µM–10 mM	1–10 mM	2.45 mM	10 mM	100 µM	100 µM
Mercury	200 nM–200 µM	2–20 µM	44 µM	2 µM	2 µM	2 µM
Thallium	10 nM–10 µM	10–100 µM	1.35 µM	10 µM	1 µM	1 µM

*Effective dose for live/dead imaging is summarized as a qualitative measure of rapid cell death detected in images. Effective dose for heart beat assay at each time point is described by a p-value of 0.05*.

### Live/Dead Staining

Organoids were isolated from 96-well low adhesion round bottom plates, suspended in Hystem hydrogel (GS311, ESI BIO, Alameida, CA, USA) in a 20 µL construct, and placed into 12-well plates to immobilize organoids in a 3D extracellular matrix environment, as we have described previously (24, 26, 27). Each concentration of environmental toxin premixed at 1X concentration in media according to organoid type was added to individual wells and organoids were allowed to incubate under their respective conditions for 2 days at 37°C with 5% CO_2_. Studies were performed using *n* = 5 or higher for all conditions. Media was then removed and organoids were assessed by LIVE/DEAD^®^ Viability/Cytotoxicity Kit assays (Invitrogen, Carlsbad, CA, USA). Specifically, 2.0 µM calcein AM and 4.0 µM ethidium homodimer in PBS was added to each well and was allowed to incubate for 1 h. Imaging was then performed by macro-confocal microscopy (Leica TCS LSI, Leica, Wetzlar, Germany) and composite images were created with ethidium bromide red fluorescence representing dead nuclei and calcein AM green fluorescence representing live cells.

### ATP Activity Assays

Environmental toxins were added in a premix 2× concentration of 100 µL solution to each well of a 96-well plate containing an organoid with 100 µL of media and allowed to incubate for 2 days at 37°C with 5% CO_2_ with *n* = 6 or higher. Media was then removed from each well leaving 100 µL of media remaining along with the organoid. Next, 100 µL of Cell-Titer Glo Luminescent Cell Viability Assay solution, prepared according to maufacturer’s instructions (G7571, Promega, Madison, WI, USA), was added to each well along with 100 µL added to 100 µL DMEM in a Costar Black Polystrene 96-well assay plate and allowed to incubate for 10 min at room temperature shielded from light. The entire contents of the wells containing organoids were then added to Costar Black Polystrene 96-well assay plate wells and the contents were read on a Vertias Microplate Luminometer using default settings. Values were then averaged among the different groups and graphed for analysis using Graph Pad Prism© software.

### Heart Beat Assay

Fully formed cardiac organoids in the wells of a 96-well non-adhesive round bottom plate were placed under a Leica DMIL LED microscope to allow for the recording of natural beat rates in 20 s videos (*n* = 3). The plate was then returned to the 37°C, 5% CO_2_ incubator for 5 min to ensure that organoids did not experience significant temperature decreases, which can detrimentally impact beating rates. The process was repeated until all experimental subject organoids under the compound concentrations described above, but at varying time points, had been recorded. Environmental toxins were added as 2 × 100 µL concentration to each well of a 96-well low adhesion round bottom plate containing 100 µL of normal media with a cardiac spheroid, and allowed to incubate for 30 min at 37°C. The plate was then recorded, 3 organoids at a time, in the process listed above until all organoids were recorded and the plate was returned to the incubator. The process was then repeated 24 and 48 h later. The 20 s videos were then analyzed: beats were counted for each video and multiplied by 3 to scale values to beats per minute. A beat was defined as the beginning of the contractile movement of the organoid. A beat did not need reach conclusion to be counted. If multiple beating regions were observed then the beating of the largest multi-cell structure was used to calculate the beating rate.

### Statistical Analysis

The data are generally presented as the means of number of replicates ± SD. All data are graphed and analyzed for significance using a Student’s *T*-test. For ATP activity assays *p*-values were considered significant under 0.01. For beating kinetic assays *p*-values were considered significant under 0.05. Data samples were eliminated from the experimental groups if they fell outside of two SDs from the experimental group averages. Sample sizes (generally *n* = 5 or *n* = 6, depending on the experiment as described) were determined based on preliminary experiments. These sample sizes, with the typically observed SDs, allowed statistical significance at α = 0.05 with statistical power greater than 80%.

## Results

### Organoid Production and Viability

**Figure 1:**
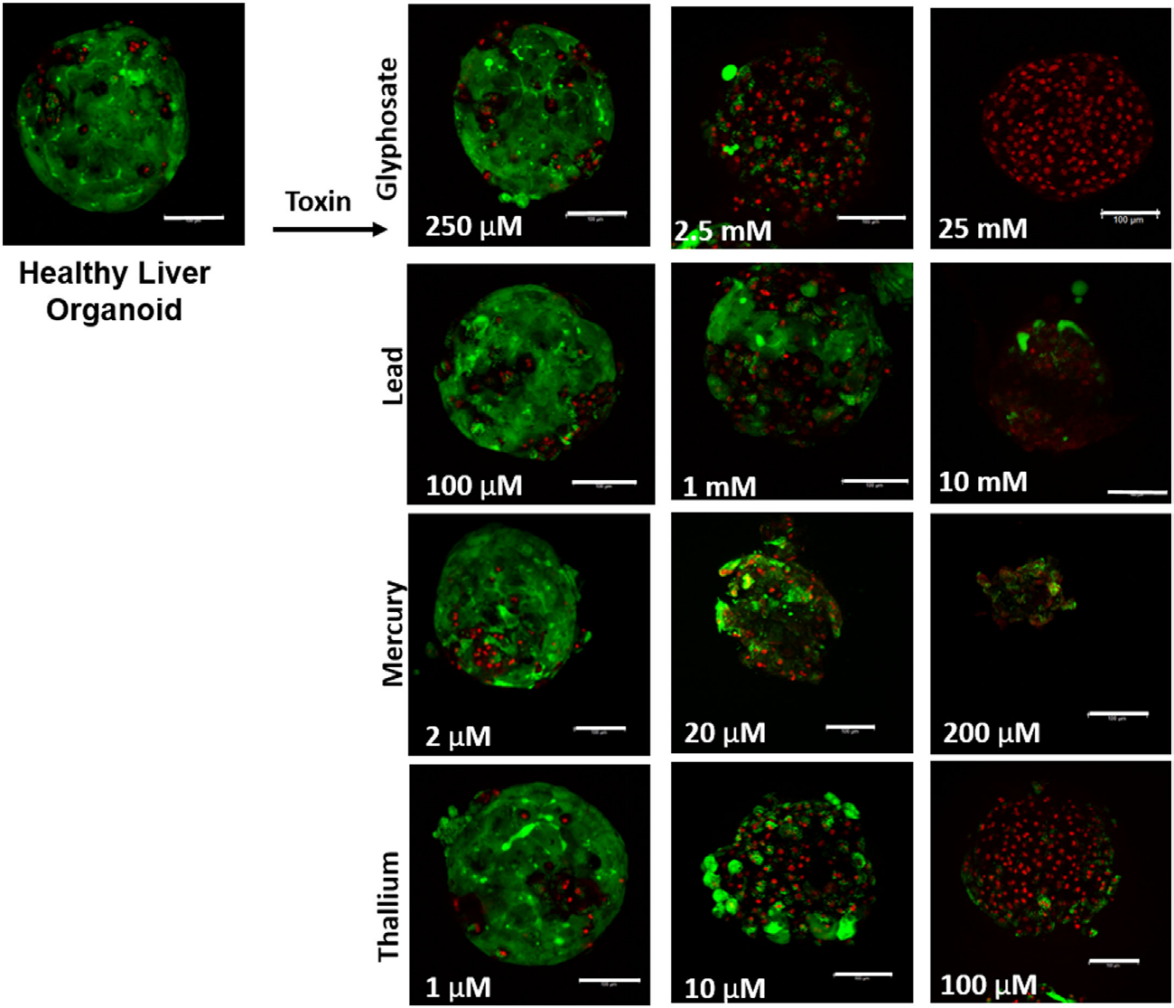
Visual assessment of environmental toxin effects on liver organoids by live/dead assay. The effects of glyphosate, lead, mercury, and thallium on liver organoids using ethidium homodimer dead (red) and calcein AM live (green) staining. Scale bars are equal to 100 µm.

**Figure 2:**
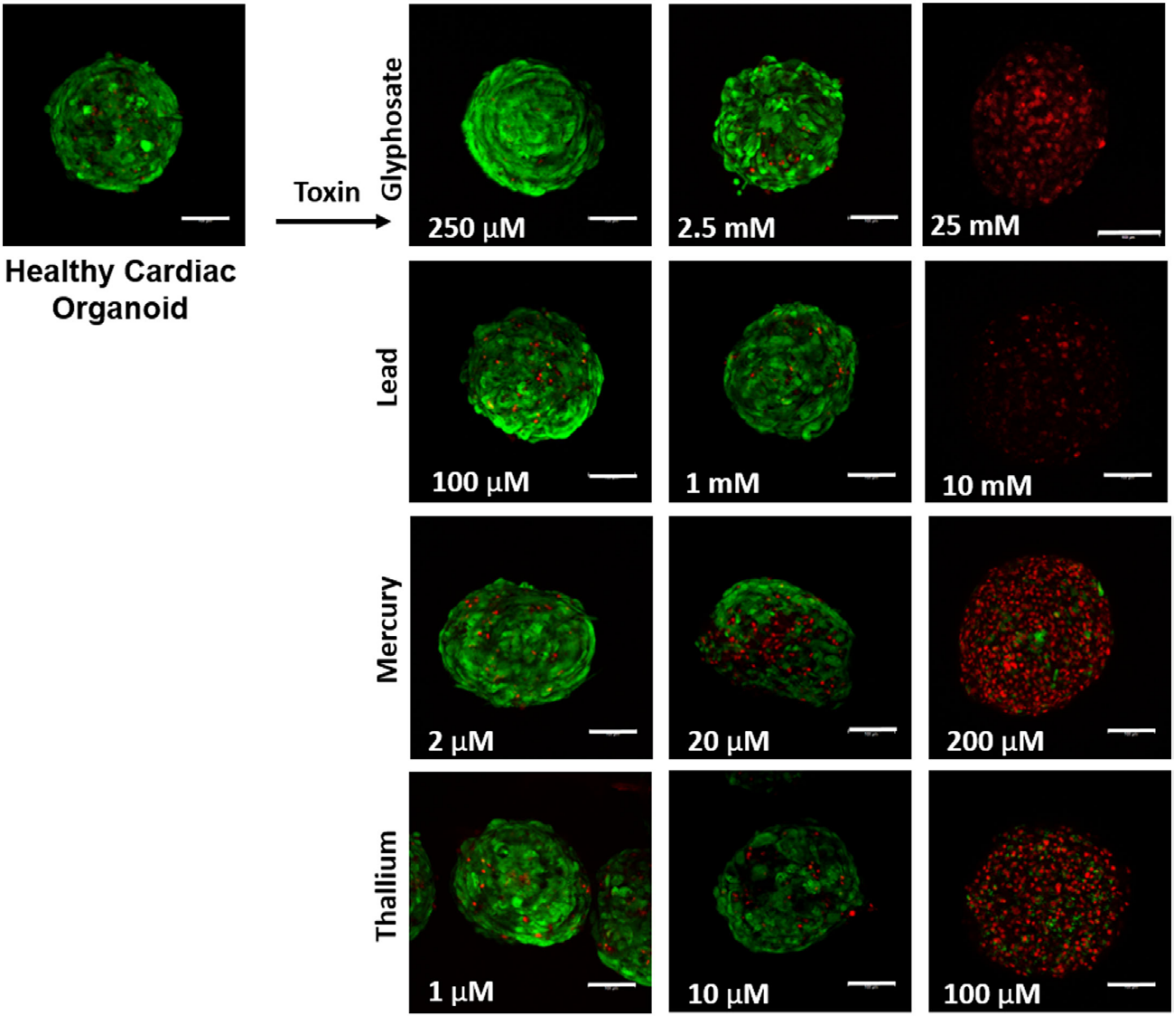
Visual assessment of environmental toxin effects on cardiac organoids by live/dead assay. The effects of glyphosate, lead, mercury, and thallium on cardiac organoids using ethidium homodimer dead (red) and calcein AM live (green) staining. Scale bars are equal to 100 µm.

Liver organoids successfully formed at 4 days, with cardiac organoids taking 7 days to allow for self-propagating beating to occur. Viability for both was confirmed to be high (greater than 95%) *via* live/dead imaging (Figures 1 and 2, top left panel).

### Environmental Toxins

Dissolution of the environmental toxins was performed in the presence of DIH_2_O due to previous research in toxin testing (28–30). Mercury (II) chloride (254.19 mM at 20°C H_2_O) and thallium (I) nitrate (358.51 mM at 20°C H_2_O) dissolved readily in DIH_2_O and remained dissolved with no precipitation during storage. Glyphosate (70.98 mM at 25°C H_2_O) did not dissolve readily until constant stirring and heat was applied, it remained dissolved at 25 mM in DIH_2_O with no precipitation after storage. Lead (II) chloride dissolved readily in DIH_2_O at 20 mM (35.60 mM at 20°C H_2_O); however, after addition to media to create 10 mM treatment some precipitate was observed, although this precipitate did not occlude LIVE/DEAD imaging.

### Organoid Viability Following Toxin Exposure

Live/dead staining of liver organoids (Figure 1) was used to visualize indications of cytotoxicity due to toxin exposure. Organoid integrity and viability begins to show a steady reduction at doses of 250 µM to 2.5 mM for glyphosate (11 mM ATP IC50), 1–10 mM for lead (2.5 mM ATP IC50), 2–20 µM for mercury (33 µM ATP IC50), and 1–10 µM for thallium (18 µM ATP IC50). Cardiac organoid responses (Figure 2) tended to occur at higher doses than displayed for liver and were more in line with IC50’s revealed by ATP testing, described below. The organoids maintained integrity during all testing, but cell death occurred between doses of 2.5 and 25 mM for glyphosate (12.1 mM for ATP IC50), 1 and 10 mM for lead (2.2 mM for ATP IC50), 20 and 200 µM for mercury (37.4 µM for ATP IC50), and between 10 and 100 µM for thallium (1 µM for ATP IC50).

### Effects of Toxin Exposure on Organoid ATP Activity

**Figure 3:**
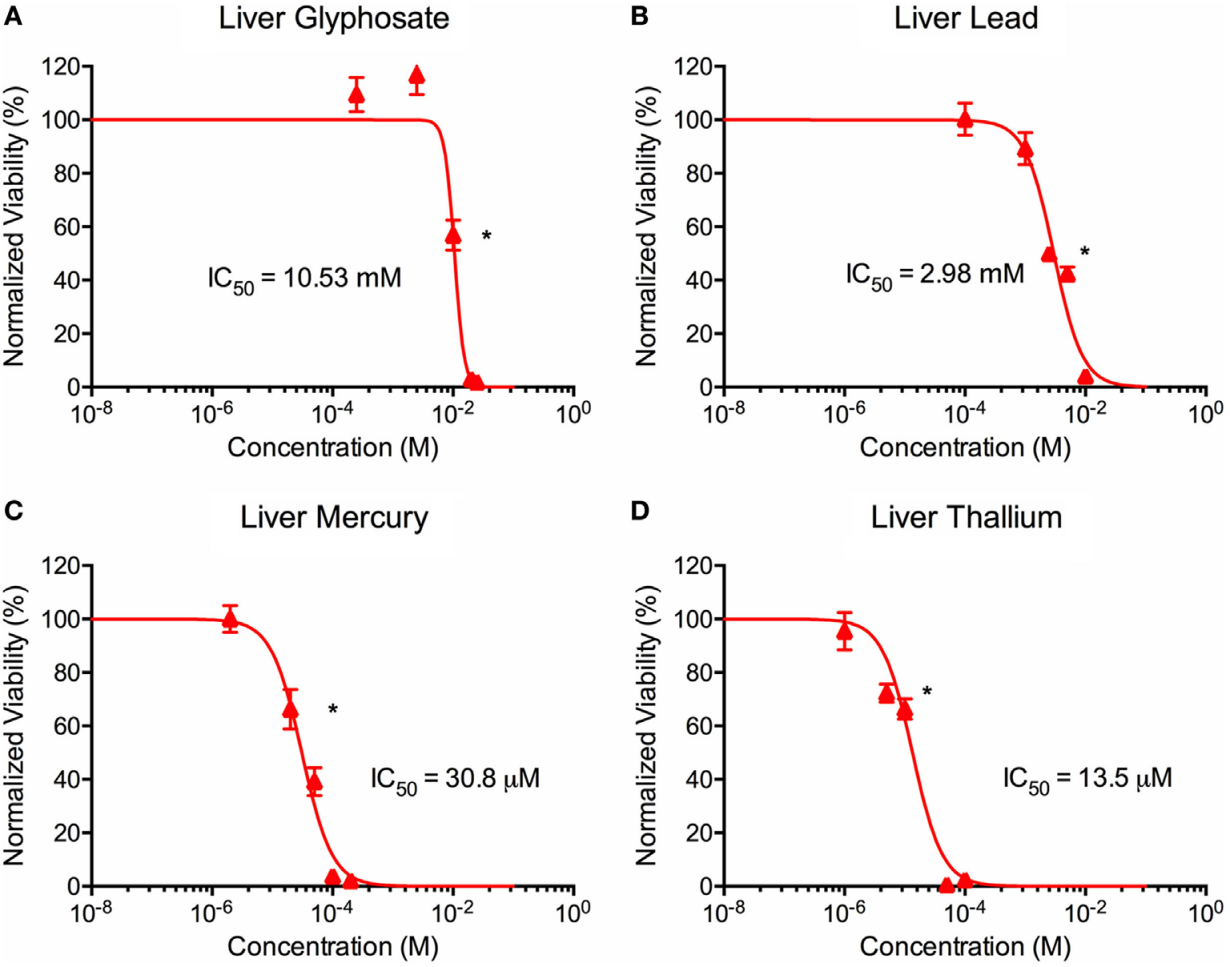
Environmental toxin effects on liver organoid ATP activity. ATP survival curves for liver organoids exposed to glyphosate **(A)**, lead **(B)**, mercury **(C)**, and thallium **(D)** over the course of two days. Adjusted ATP activity is a comparison of the ATP value of organoids exposed to toxins over the control liver organoids. Statistical significance: *p < 0.01 with bracket encompassing all doses with this value. IC50 value was determined through calculation of line crossing the IC50 marker.

**Figure 4:**
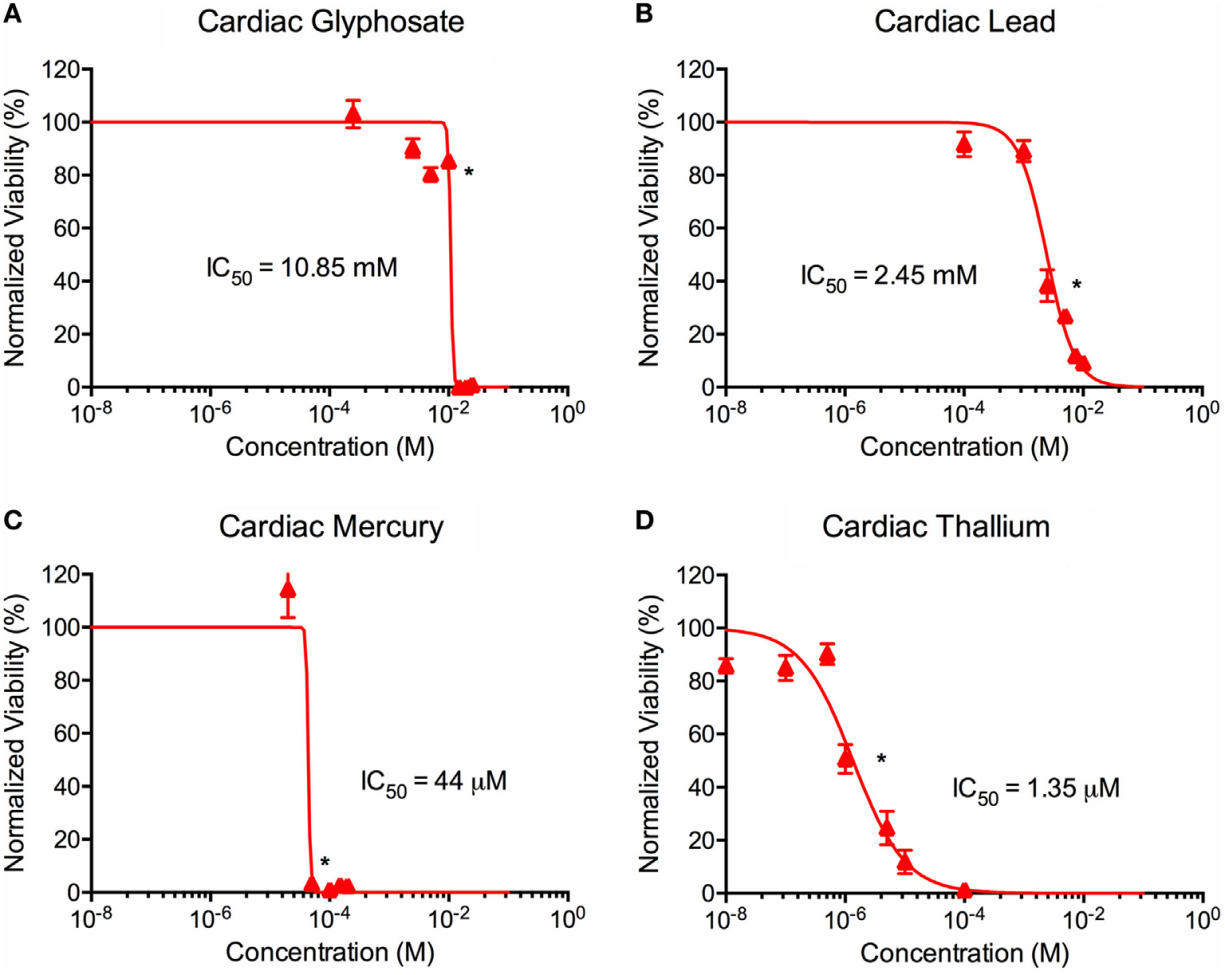
Environmental toxin effects on cardiac organoid ATP activity. Survival curves for cardiac organoids exposed to glyphosate **(A)**, lead **(B)**, mercury **(C)**, and thallium **(D)** over the course of two days. Adjusted ATP activity is a comparison of the ATP value of organoids exposed to toxins over the control cardiac organoids. Statistical significance: *p < 0.01 with bracket encompassing all doses with this value. IC50 value was determined through calculation of line crossing the IC50 marker.

ATP activity test in this experiment was designed to determine the dose for IC50 value for each of the environmental toxins on primary liver (Figure 3) and cardiac (Figure 4) organoids and are summarized in Tables 1 and 2. After testing original three doses (*n* ≥ 6) for each toxin centered on doses described in the literature (28, 29), two further trials narrowed the range containing the IC50. IC50 was calculated using the Graph Pad Prism© software. IC50 numbers were similar for cardiac and liver for three of the drugs: glyphosate (10.85, 10.53 mM), lead (2.45, 2.98 mM), and mercury (44, 30.8 µM), respectively. Thallium toxicity was determined as 1.35 and 13.5 µM for liver and cardiac, respectively. For liver organoids, the following conditions resulted in statistically significant decreases in ATP activity (*p* < 0.001): 10 mM glyphosate, 2.5 mM lead, 20 µM mercury, 5 µM thallium, and higher concentrations. For cardiac organoids, the following conditions resulted in statistically significant decreases in ATP activity (*p* < 0.001): 5 mM glyphosate, 2.5 mM lead, 50 µM mercury, 1 µM thallium, and higher concentrations. Significant *p*-values were detected at higher than control value for the lowest values of mercury doses tested, 2 and 10 µM, reflecting results observed for heartbeat assay with an increase at 20 µM short term (Figure 4C).

### Toxin Exposures Decrease Cardiac Organoid Beating Rates

**Figure 5:**
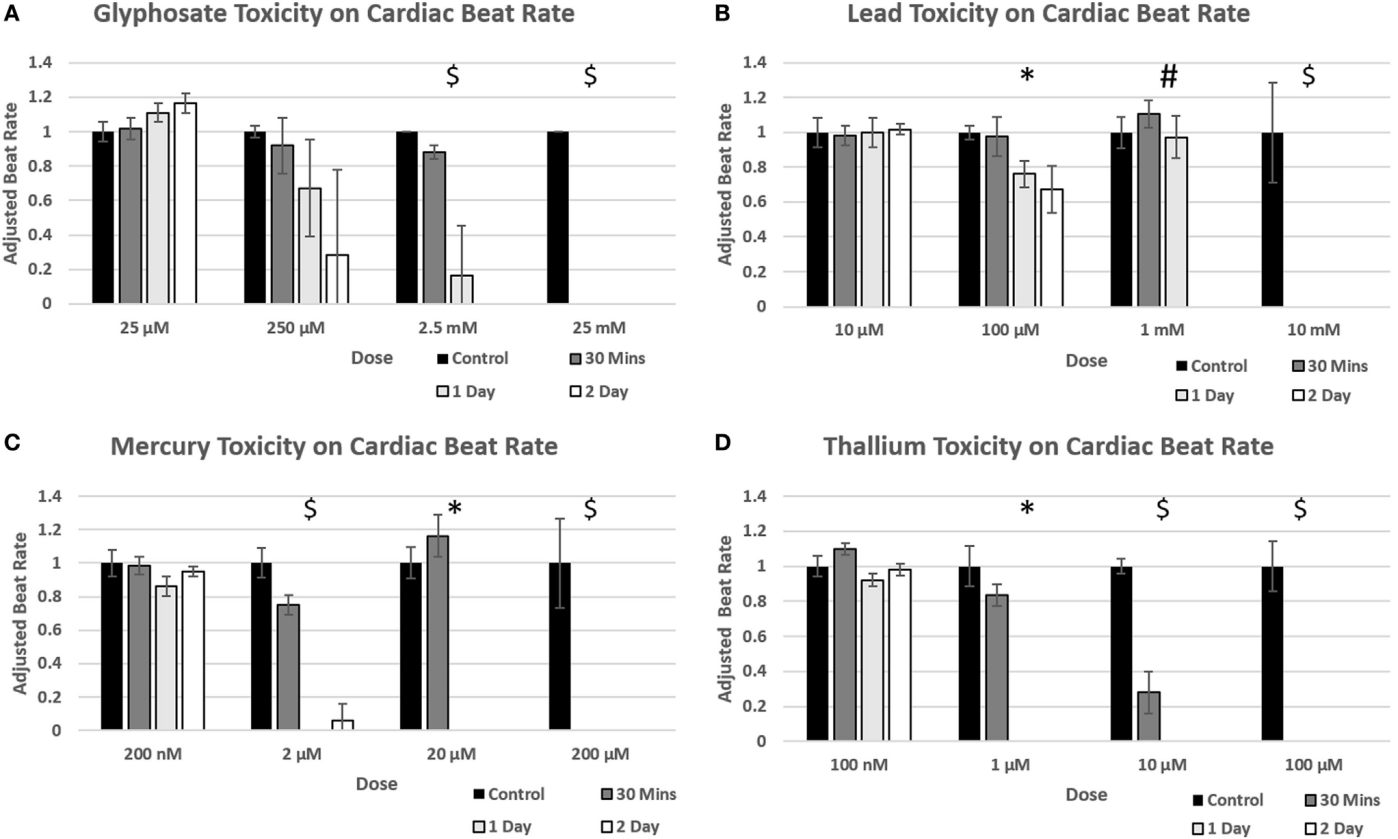
Environmental toxin influence on cardiac organoid beating kinetics. Figure 4 demonstrates the effect of glyphosate **(A)**, lead **(B)**, mercury **(C)**, and thallium **(D)** on cardiac organoid beat rate. All values (*n* = 3) were taken over 20 s videos and multiplied by three to find per minute. The adjusted rate is calculated by taking average value of each condition and dividing over the control average. A *T*-test was performed to determine significance between control rates and time after drug was added. Statistical significance: #*p* < 0.05 at 2 days; *p < 0.05 at 1 day; $*p* < 0.05 at 30 min.

Time points to test physiological reactions to environmental toxins were chosen based on previous studies testing calcium channel impairment in 2D cardiomyocyte populations and defined as immediate (30 min), short term (1 day), and long term (2 days). *p*-values below 0.05 were defined as significant. Glyphosate demonstrated a range of toxicity at doses of 250 µM and higher, with beat rates at 250 µM slowing on day one among all organoids and two of the three organoids ceasing beat on day 2, although this did not reach a significant *p*-value due to high SD of sample (Figure 5A). At 2.5 mM, 1-day toxicity caused two spheroids to cease beating entirely, with one-third showing a 50% reduction in beat rate; by day 2 all had ceased beating. Lead toxicity presented at 100 µM with significant beat reduction at 1 day. 1 mM lead did not display a significant beat reduction at 1 day, but by 2 days showed a complete cessation (Figure 5B). Mercury was highly effective at stopping beat rate over the course of 24 h, with doses of 2 µM and above ceasing beating. Of the 2 µM, one organoid ceased beating on day 1 and began to slowly beat on day 2, the only organoid in the entire study to do so (Figure 5C). A rise in beat rate was observed at 20 µM, reflecting a result shown in ATP assay of raised ATP at doses of 10 and 20 µM before falling to IC50 value (Figure 4C). Thallium demonstrated toxic effects at all concentrations of 1 µM and above, in particular at concentrations of 10 µM with severe depressive effects on heart beat at 30 min and total cessation of all time points at concentration of 100 µM (Figure 5D).

## Discussion

While countless environmental toxins exist, and there are many that are pertinent to human health, and many more that require study to understand their potential impacts on human health, we chose several traditional, well-studied toxins to challenge our organoid systems. Our objective was to display the utility of bioengineered 3D *in vitro* organoids for rapid environmental toxin screening, using multiple widely studied toxins that have been explored thoroughly in animal models and for which toxicity in humans has been also well documented. Specifically, testing began with well-researched heavy metal and pesticide toxins due to the significant amount of past research into each of them, albeit often in 2D cell culture systems. As such, the doses for original testing were gathered from literature as a starting point for our own calculations (28–30). As all have shown toxic effects on multiple organ systems in the body, our system comprising organoids representing multiple tissue types can be utilized to show both general and specific toxicity toward each of the tissue organoids present, encompassing metrics derived from ATP activity assays, LIVE/DEAD-stained organoid images, and physiological heart beat assays. From these tests, we can demonstrate that our organoids begin to show observable physiological effects in a time frame that is generally faster, and by methods less time intensive and complicated than probing of biochemical markers, as well as being one of the first documented deployments of 3D organoid models for detection of these acute toxicities for environmental toxins. Combining the results of these tests creates a picture of toxicity that demonstrates the value of multiple tests to show the progression of toxicity in our organoids, with the ideal goal being a comparison to those levels found in human blood plasma to show our system effectively mimics human toxicity.

Three well known environmental toxins and a pesticide were selected for testing in both the cardiac and liver model systems: glyphosate, lead, mercury, and thallium. Glyphosate (*N*-phosphonomethyl glycine) is used as a part of pesticides on crops that have been bred to have resistance to it; however, the chemical is present on crops after harvest. Glyphosate has been found to negatively affect endocrine receptors in the body and is toxic to liver hepatocytes both *in vivo* and *in vitro* (31). It has also been potentially linked to a wide range of ailments, including diabetes, cancer, stroke, and Parkinson’s disease (15). Lead toxicity manifests itself in a multitude of ways, including increasing free radical oxygen species causing oxidative stress by affecting the creation and efficacy of antioxidants created from glutathione by displacing sulfide groups, decreasing the reduced form and increasing the oxidized form, and causing an increase in reactive oxygen species (ROS) (32). Additionally, lead can also displace other ions with a +2 charge, including Ca^+2^, Mg^+2^, and Fe^+2^ that are involved in cellular processes and affect both efficiency and create direct and indirect cytotoxicity. Mercury bioaccumulation effects have been well researched in marine life and affect humans through consumption. Its effects on the neurological system are best understood, but it can affect any organ which is exposed through several mechanisms, including binding available thiols, interrupting Ca^2+^ homeostasis through membrane potential changes, and affecting the creation and stability of several cellular components (33). In cardiac tissue, it can affect myosin ATPase and myocardial force development. Thallium toxicity is most often associated with substitution for K^+^ ions by cellular membranes due to similar charge and the negative effects this has on the Na/K pump (34, 35). Further research has also shown that thallium can directly affect membrane integrity in multiple organelles, including mitochondria, and bind open sulfyhydrol sites in enzymes (36–38). After its accidental discovery, thallium was used as a pesticide and rodenticide as well as a medical treatment of various ailments until it was banned in the US due to risk of accidental poisoning (39).

Our experiments displayed lead toxicity in both cardiac and liver organoids with an ATP IC50 of 2.45 and 2.98 mM, respectively, and a significant reduction of heartbeat at 2 days for 100 µM. Lead exposure typically occurs through either ingestion or inhalation and sources of exposure to humans are numerous (40). In Tchounwou et al., an IC50 of 113 µM was found for a 2D incubation of HepG2, however, the review of cells present (500,000 per well) is at a significantly higher scale than our experiments. Many other studies have focused on long-term animal studies and physiological differences in cell populations (41–44). Previous research on levels of lead in blood plasma that can lead to fatal outcomes has shown 14.47 µM in humans, lower than both our research values and other *in vitro* models. The probable reason is that lead toxicity often manifests as a prolonged, low exposure systemic poisoning affecting many systems in the patient; it would be unlikely that a person would be exposed to enough lead to cause acute symptoms (45–47). Our research has displayed the system will be able to detect toxicity in this type of exposure if it were to present itself.

Mercury toxicity was displayed at an ATP IC50 of 30.8 and 44 µM for liver and cardiac organoids, respectively, significant cell death for both at 20 µM in live/dead imaging and ceased heartbeats after 1 day at doses of 2 µM and higher. Mercury has been long understood as a toxin with effects on a majority of the systems in the body, including liver and cardiac function (48, 49). One study comparing *in vitro* liver toxicity over 24 hours between several cell types in 2D, including primary human hepatocytes (106 µM), primary rat hepatocytes (726 µM), the HepG2 cell line (48 µM), and the mouse 3T3 fibroblast cell line (8 µM) to estimated lethal plasma levels in humans (6.5 µM) compared well to our calculated values (30.8 and 44 µM for liver and cardiac ATP, respectively and 2 µM for affecting heart rate) (50).

Thallium was the only toxin with vastly different ATP IC50 doses between cardiac and liver organoids (1.35 and 13.5 µM, respectively), and stopped heartbeats at 1 day of exposure at 1 nM. Similar to mercury, the study comparing *in vitro* liver toxicity over 24 h between several cell types in 2D, including primary human hepatocytes (1,152 µM), primary rat hepatocytes (85 µM), HepG2 cell line (1,266 µM), and mouse 3T3 fibroblast cell line (1,527 µM) to estimated lethal plasma levels in humans (2.33 µM) displayed a large difference between the values, suggesting the acute toxicity is not due to hepatic-related mechanisms and may be related to a physiological effect instead of general cellular toxicity (50). While our IC50 for primary liver cells is higher than the lethal patient plasma level in this experiment, the cardiac value for our ATP IC50 (1.35 µM) is similar to this value, with the dose affecting heart beat rate at the same dose. A disruption in K/Na channel function would affect beat rate significantly and explain the loss of beat rate in relatively low doses tested. Another study on Jurkat cells, immortalized T lymphocytes, in 2D determined toxicity at 500 µM, well above the toxicity calculated by our experiment (51), although histological liver damage was detected in rats exposed to thallium (52). Other studies on the patient plasma levels of thallium required for lethality have shown a range from 2.44 to 53.82 µM. Our toxicity values for both liver and cardiac organoids are encapsulated within this range.

Glyphosate live/dead imaging varied greatly, with the 2.5 mM showing significant cell death in liver organoids and little cell death in cardiac organoids. However, ATP values taken at 10 mM showed a large difference for both the liver and cardiac organoids (0.568 and 0.804, respectively), displaying a more gradual decrease in cell viability for the liver organoids as compared to the cardiac organoids, especially when taking into account the similar IC50 values for the two (10.5 and 10.8 mM, respectively), suggesting a variance in toxicity point between the organoids for cardiac, especially when taking into account the differences in beating rate stoppage among the 0.25 and 2.5 mM cardiac organoids. Previous work to determine hepatotoxicity of glyphosate determined an IC50 value of 37.9 mM on a 2D HepG2 model, displaying our 3D primary system as significantly more sensitive (28). Other experiments utilizing Roundup©, the common herbicide utilizing glyphosate as an active ingredient, demonstrate a range of effects on cellular components, ranging from membrane swelling in multiple organelles to increased lysosome numbers and effects on nuclear transcription along with causing hormonal issues in mammalian testing subjects (53–55). Although these effects may be attributable to other ingredients present in the formulation, current opinion remains that continued use of glyphosate in pesticides should be reviewed (53).

By utilizing multiple viability tests, we are able to detect levels of toxicity in multiple assays when exposed to environmental toxins present in the system. Our system is capable of measuring the acute effects on both liver and cardiac 3D organoids and would be easily adaptable to any newly developed organoid, newly discovered toxins, or extended time length. Additionally, it would be straightforward to add additional test protocols, as we have employed in other drug interactions studies (24), to create a more complete picture of toxicity on our representative human organoids. Importantly, organoid platforms such as these, which are 3D, derived from human cells, and exhibit high levels of functionality and physiologically accurate responses to external stimuli, offer a powerful technology that can be deployed for environmental toxin screening, as well as other applications, such as drug development, diagnostics, and personalized medicine.

## Author Contributions

SF, MD, TS, and AS designed the experiments. SF performed the majority of organoid toxicity studies. MD performed all confocal microscopy. CB generated the liver and cardiac organoids. SF, TS, SS, AA, and AS evaluated the data and interpreted the results. SF, MD, SS, AA, and AS wrote and edited the manuscript.

## Conflict of Interest Statement

The authors declare that the research was conducted in the absence of any commercial or financial relationships that could be construed as a potential conflict of interest.
